# From classrooms to cross-borders: early childhood educator preparation in the Philippines and its influence on migration decisions

**DOI:** 10.3389/fsoc.2025.1643165

**Published:** 2025-12-09

**Authors:** Nilo Jayoma Castulo, Arlyne C. Marasigan, Heidi B. Macahilig, Nikolee Marie A. Serafico-Reyes, Esayas Teshome Taddese

**Affiliations:** 1 Department of Educational Leadership and Professional Services, College of Education, Mindanao State University – Tawi-Tawi College of Technology and Oceanography, Bongao, Philippines; 2 Faculty of Behavioral and Social Sciences, and Educational Policy Research and Development Office, Philippine Normal University, Manila, Philippines; 3 Faculty of Education and Liberal Arts, INTI International University, Nilai, Malaysia

**Keywords:** early childhood education, education quality, teacher migration, Philippines, professional identity, migration intention, push-pull migration theory, EDCOM 2

## Abstract

**Introduction:**

The Philippines faces significant challenges in its Early Childhood Education (ECE) sector, including issues of quality, teacher preparation, and the growing trend of educator migration, which threatens the sustainability of the workforce. This study explores the current status of Philippine Early Childhood Education, including its systemic challenges in ECE teacher preparation, and the influence of migration intentions among ECE stakeholders across selected Philippine Teacher Education Institutions (TEIs).

**Methods:**

A qualitative case study design was employed, involving 40 key informant interviews and focus group interviews (administrators, cooperating teachers, faculty members, and pre-service teachers) across five state-funded Philippine Teacher Education Institutions (TEIs) designated as Centers of Excellence. Data were analyzed thematically using the push-pull migration theory and professional identity theory.

**Results:**

The findings showed that ECE stakeholders perceived low programme quality, limited career advancement, financial instability, inadequate institutional support, and societal stigma. Systemic challenges in ECE teacher preparation include real-world misalignment of the curriculum, resource limitations, insufficient training and support, and administrative challenges. Factors that influence migration intention include higher compensation, demand from recruitment agencies, better opportunities abroad, family sacrifices, and educational and professional development.

**Discussion:**

This study showed that migration is both a response to systemic inequalities and an expression of professional autonomy. Furthermore, we investigated effective strategies for retaining ECE teachers in comparable countries within the Global South to gain transferable insights. Although the study’s findings cannot be universally applied to the entire context of the Philippines, they provide valuable insights into the realities of ECE teacher demand and supply, as well as the challenges faced domestically.

## 1 Introduction

The challenges faced by higher education institutions and various forms of illiteracy are rooted in fundamental aspects of human development. The early years are a crucial period for developing essential skills, such as self-regulation and emotional competence. Educators who can manage their emotions and stress are more effective in fostering these abilities in children (Housman, 2021). Effective training of teachers in early childhood education is vital to enhance their professional expertise, improve the quality of educational programs, and promote positive outcomes in children’s cognitive, social, and emotional development (Alstad, 2022; Dunekacke et al., 2021; Ledoux et al., 2008). Research shows that high-quality early childhood education programs led by skilled educators provide lasting benefits for children’s cognitive and social growth (Ledoux et al., 2008). A study examining early childhood teacher preparation programs found differences in student teaching experiences, with 4-year programs generally offering more extensive opportunities than 2-year programs (Sumrall et al., 2017). Existing literature highlights a significant gap in studies focused on preparing cooperating teachers to provide quality field experiences for future educators (Baum and Korth, 2013). While many programs incorporate this content into their mission, there is notable variation in coursework and practicum experiences, underscoring discrepancies in preparing educators to support children with disabilities (Muthukrishnan et al., 2024; Chang et al., 2005).

A thorough investigation is essential to establish a solid basis for evaluating the effects of teacher education on early childhood education (ECE) outcomes. It is essential to identify individuals and ways in which teacher education is important (Horm et al., 2013). Empirical studies highlight the importance of developing innovative course designs and gaining more profound insight into the knowledge, beliefs, and practices of both preservice and in-service educators (Alstad, 2022). Additional inquiry is necessary to delve into these domains and to develop impactful training initiatives. Collaboration and cross-cultural studies among early childhood education professionals from other nations should be encouraged to improve the quality and status of such education (Al-Hassan, 2020). This approach has the potential to provide valuable insights into effective practices and improve teacher training globally. There is a need for increased professional standards in early childhood education, which encompasses more rigorous selection processes for candidate teachers, improved compensation for educators, and a more robust curriculum (O’Brien, 2015). Filling these gaps can contribute to attracting and retaining high-quality educators in the field. Thus, this study explores the current status of Philippine Early Childhood Education, including its systemic challenges in ECE teacher preparation and the influencing migration intentions among ECE stakeholders across the selected Philippine TEIs. This study seeks to answer the following research question:

1. What are ECE stakeholders’ perceptions of ECE quality and their professional identity across the selected Philippine TEIs?
2. What systemic challenges in ECE teacher preparation among pre-service ECE teachers shape migration considerations?
3. Based on these systemic challenges and perceptions of ECE quality, what made them contribute to their migration intention to leave the Philippines?

## 2 Literature review

### 2.1 Research context of Filipino early childhood education and ECE teachers’ migration

The first 1,000 days of a child’s life are crucial for their development, and adequate nutrition during this period is essential for their growth and success. Nevertheless, malnutrition remains a significant problem for young children in the Philippines despite numerous efforts to address it. This ongoing issue continues to impede the national development of children in the nation (Silva et al., 2024). Thus, the role of Early Childhood Care and Development (ECCD), ‘significant others’ are crucial in fostering the comprehensive growth of children, aiding in their health, education, and social interactions who extend beyond parents and guardians encompass grandparents, members of the extended family like aunts, uncles, and cousins, as well as yayas (nannies), Kasambahays (household helpers), community health workers, educators, and religious figures (Lasco and Mendoza, 2025). Unfortunately, in the Philippines, Early Childhood Education has not been formally established, supported, or integrated with higher education. Instead, it is primarily the responsibility of local governments rather than being a key area for development as a solid foundation. The sluggish progress in early childhood care and development (ECCD) in the Philippines has been attributed to multiple factors (Second Congressional Commission on Education, 2024, 2025). Cultural beliefs regarding child development contribute to the notion that early education is not essential, which is further exacerbated by a lack of awareness of its advantages. The government’s insufficient focus and inadequate infrastructure limits participation, with private schools being an option only for those who can afford them. Financial constraints prevent families, especially in low-income areas, from accessing formal day care and educational services. Underinvestment in ECCD prioritizes short-term health measures over long-term educational goals (Ulep et al., 2024).

Table 1 presents the chronological development of the Philippine Early Childhood Care and Development (ECCD) policy through a combination of national laws, Department of Education (DepEd) Orders, and Commission on Higher Education (CHED) Memorandum Orders, illustrating the country’s gradual but consistent move toward an integrated, rights-based, and developmental approach to early learning. Beginning with RA 6972 (1990), which institutionalized community-based day-care centers, the ECCD system was further formalized by RA 8980 (2000), which established a coordinated national ECCD framework for children aged 0–6 years. The passages of RA 10157 (2012) and RA 10533 (2013) marked a paradigm shift toward compulsory and quality kindergarten education, reinforced by the DepEd Omnibus Policy on Kindergarten (DO 47, 2016). The CHED CMO 76, s. In 2017, this was complemented by setting national standards for pre-service Early Childhood Education teacher preparation. More recent policy reforms, such as RA 12199 (2025), have replaced RA 10410 (2013) to strengthen inter-agency collaboration between the ECCD Council and DepEd, ensuring inclusion, health, and nutrition integration in early education. This evolution underscores the Philippines’ efforts to align ECCD with Sustainable Development Goals by linking early learning with health, nutrition, and teacher quality reforms, as emphasized in the Philippine Institute for Development Studies’ policy analysis, which notes that early education and proper nutrition during the first years of life are crucial to improving learning outcomes and long-term development prospects for Filipino children (Ulep et al., 2023).

**Table 1:** Timeline of ECCD-related legal and policy instruments in the Philippines.

Year	Instrument	Key policy content / ECCD relevance
1990	Republic Act (RA) 6972—Barangay-Level Total Development and Protection of Children Act	Requires every barangay to establish a day care center for children aged 0–6; provides integrated services for health, nutrition, and early education at the community level.
2000	RA 8980—Early Childhood Care and Development (ECCD) Act	Institutionalizes a National ECCD System for children 0–6 years old; mandates coordination among DepEd, DSWD, DOH, and local governments; sets curriculum standards for early childhood care and education.
2001	RA 9155—Governance of Basic Education Act	Defines DepEd’s governance structure; decentralizes management of basic education, including ECCD and Kindergarten, through regional and division offices.
2012	RA 10157—Kindergarten Education Act	Makes Kindergarten education compulsory and a prerequisite to Grade 1; mandates that all 5-year-old children must undergo one year of Kindergarten under DepEd supervision.
2012	DepEd Order (DO) No. 32, s. 2012—*Institutionalizing Kindergarten Education*	Implements RA 10157; provides transitional guidelines for the universal implementation of Kindergarten; clarifies curriculum and teacher qualifications.
2013	RA 10410—Early Years Act (EYA)	Recognizes 0–8 years as crucial to development; assigns the ECCD Council to oversee children 0–4 years, and DepEd for 5–8 years; ensures alignment between early childhood and basic education programs. *(Now repealed by RA 12199)*
2013	RA 10533—Enhanced Basic Education Act (K to 12 Law)	Expands basic education from 10 to 13 years; integrates Kindergarten as the first stage; introduces learner-centered and developmentally appropriate pedagogy in early education.
2016	DepEd Order (DO) No. 47, s. 2016—Omnibus Policy on Kindergarten Education	Consolidates all Kindergarten policies; defines curriculum standards, learning environment, assessment, and teacher qualifications; anchors Kindergarten within inclusive and developmentally appropriate frameworks.
2017	CHED Memorandum Order (CMO) No. 76, s. 2017—Policies, Standards, and Guidelines (PSGs) for Bachelor of Early Childhood Education (BECEd)	Sets outcomes-based curriculum standards for ECE teacher preparation; defines competencies in pedagogy, child development, and ECCD practice; aligns with DepEd’s Kindergarten policies.
2018	DepEd Order (DO) No. 20, s. 2018—Amendment to DO 47, s. 2016 (Cut-off Age Policy)	Establishes age cut-off for Kindergarten enrollment (5 years old by June 1); provides transitional provisions for those turning 5 by August; applies to both public and private schools.
2025	RA 12199—Early Childhood Care and Development System Act	Repeals RA 10410; further strengthens the ECCD System; clarifies the ECCD Council’s authority for ages below 5, and DepEd’s for ages 5–8; institutionalizes inter-agency collaboration on inclusion, health, and nutrition.
2025	DepEd Order (DO) No. 15, s. 2025—Amendment to DO 47, s. 2016	Updates the Kindergarten cut-off age to October 31, repealing DO 20, s. 2018; provides greater flexibility for enrollment and alignment with ECCD transitions under RA 12199.

Sources: Ched Memo Order No. 76 (2017); Dep Ed Order No 32 (2012); DepEd Order No. (2025); DepEd Order No. 20 (2018); DepEd Order No. 47 (2016); Republic Act No. 6972 (1990); Republic Act No. 8980 (2000); Republic Act No. 9155 (2001); Republic Act No. 10157 (2012); Republic Act No. 10410 (2013); Republic Act No. 10533 (2013); Republic Act No. 12199 (2025).

### 2.2 Systemic challenges in ECE teacher preparation in the Philippines and beyond

Research indicates that Filipino teachers are employed across various regions worldwide to address teacher shortages, while simultaneously managing the surplus of Filipino graduates in the Philippines (De Oca and Malaga, 2025; Docot, 2025; Lowe et al., 2016; Tsang and Lowe, 2018). According to Pacala (2024), the number of Filipino teachers in Uzbekistan is increasing, and these educators are demonstrating resilience and adaptability in their new surroundings. Jacela’s (2025) study highlights that Filipino preschool teachers encounter numerous challenges, such as adapting to local culture and curriculum, overcoming language barriers, and facing difficulties in teaching special-needs children within a different cultural context. The Filipino community engaged in the English Language Training (ELT) sector in China has faced significant challenges due to sudden policy changes by the Chinese government, which have restricted private tutoring services and foreign investment. These developments have posed substantial difficulties for Filipino teachers in China, highlighting the racial and linguistic hierarchies that affect their labor market conditions (Docot, 2025). Additionally, Filipino educators are in demand in privately operated international schools in Indonesia (Tsang and Lowe, 2018). Many educators experience unstable employment situations, often arriving without guaranteed work visas. Despite these challenges, they cultivate a spirit of global interconnectedness and aim to leverage their experiences to seek better opportunities in various contexts. Filipino educators are moving to the United States primarily in search of improved living standards and greater financial opportunities (Tsang and Lowe, 2018).

In the Philippines, early childhood educators (ECTs) encounter significant gaps between the requirements for aiding children with autism and the resources at their disposal, underscoring the need for focused professional development and systemic backing (Kim et al., 2024). The country’s education system struggles with high student-to-teacher ratios, shortage of educators, insufficient resources, low pay, and systemic corruption, all of which create a challenging setting for effective teacher training (Chua, 2019). Public education often faces interruptions owing to policy shifts, inadequate infrastructure, technological limitations, and climate-related emergencies. To address these challenges, sustainable programs that emphasize adaptability and responsiveness are crucial (Delgado et al., 2021). The success of early childhood education is heavily affected by family engagement, cultural factors, and community connections, highlighting the need for effective administrative models to enhance practices (Lubrica et al., 2012). The difficulties in preparing ECE teachers are not exclusive to the Philippines, as low- and middle-income countries (LMIC) also face similar issues, such as limited resources, insufficient training, and the necessity for sustainable strategies to improve workforce quality (Pearson and Siraj, 2025). In the Philippines, the Early Childhood Care and Development Council (ECCD) has established specific standards for cultural, linguistic, and indigenous competencies for ECE teachers (Biana et al., 2021). However, there remains a need for additional training to create an inclusive curriculum that better accommodates cultural diversity (Biana et al., 2021).

In several Asian nations, educators encounter the challenge of balancing diverse and occasionally opposing cultural and educational perspectives, a situation exacerbated by globalization’s impact on local policies and practices (Gupta, 2014). There is a worldwide demand for professional development initiatives that improve teacher skills, especially in areas such as music education, which is frequently overlooked in teacher-training programs (Bautista et al., 2024; Cheng et al., 2024). The decentralized structure of early childhood education systems, along with high rates of teacher turnover, results in low entry standards and relaxed regulations, which undermine the quality of education (Cho and Couse, 2008). Systemic societal inequalities disproportionately limit children’s access to early learning settings, highlighting the need for policies and practices that foster equity and inclusion (Friesen et al., 2024). While global studies highlight migration drivers among early childhood teachers, empirical evidence from the Philippines remains sparse, particularly regarding how teacher preparation influences migration intentions.

### 2.3 Studies on early childhood education teacher preparation

In the United States, research on early childhood teacher education preparation has concentrated on enhancing the intercultural competence of preservice teachers through a variety of field experiences, both within the country and abroad. These programs have demonstrated notable advancements in teachers’ sensitivity to intercultural issues and in their capacity to value cultural diversity (Lash et al., 2022). Institutions place strong emphasis on language, literacy, child development, and interactions between adults and children. The methods of supervision and evaluation tools differ, with some programs adopting published tools and others adhering to local or national standards (La Paro et al., 2014). These programs strive to align themselves with national standards and prioritize reflective practices. The implementation of electronic portfolios and standardized courses has been investigated to ensure effective teaching practices (Ledoux and McHenry, 2006; Scott-Little et al., 2011). In Spain, teacher training has been adapted to meet both national and European standards, focusing on the pedagogical needs of rural areas. Strengths, Weaknesses, Opportunities, and Threats (SWOT) analyses have pinpointed areas that need improvement, such as curriculum design and ongoing education (Gutierrez, 2019; Valle-Flórez et al., 2024). In Finland and Estonia, the Early Childhood Classroom Observation Measure (ECCOM) has been validated as a reliable tool for evaluating classroom quality, supporting its application in teacher education and professional development (Lerkkanen et al., 2012). In Chile, initial teacher training focused on social cohesion and sustainable development. While these training programs are perceived positively, there is still potential for enhancing institutional policies and teacher involvement (Concha-Díaz et al., 2024).

Countries such as India, Singapore, China, Sri Lanka, and the Maldives demonstrate a mix of native and colonial influences on their early childhood education (ECE) policies and practices. These nations aim to harmonize international and local educational perspectives, focusing on play-based and child-centered teaching methods, curriculum development, and imparting values (Gupta, 2014). In Malaysia, the government has set a goal of elevating the minimum qualification for preschool teachers to a diploma by 2020. Although many private sector educators are underqualified and underpaid, they are willing to enhance their skills if given the chance (Foong et al., 2018). India’s National Education Policy 2020 seeks to enhance ECE quality, while Bangladesh faces challenges due to a shortage of trained teachers and insufficient professional development. Insights from India’s policy could potentially improve ECE programs (Dasgupta, 2025). In Jordan, ECE teacher education faces obstacles, such as academic and financial challenges, issues with field training, and the overall status of early childhood education. Recommendations include conducting further research and cross-cultural studies to improve teacher preparation (Al-Hassan, 2020). In Ghana, research has emphasized the necessity of transitioning from traditional teaching methods to more inclusive practices. Pre-service teachers often encounter difficulties in curriculum planning and establishing their professional identity (Agbenyega and Klibthong, 2011). Studies across 22 countries have examined the impact of teacher training on ECE practices, underscoring the need for policy insights and future research to address global challenges in early childhood education (Garvis and Phillipson, 2019).

### 2.4 Theoretical underpinning

This study utilized push-pull migration theory (Lee, 1966) and professional identity theory (Beauchamp and Thomas, 2009). Complementary theories offer a deeper insight into why push factors are discouraging and pull factors are so appealing, extending beyond mere economic reasons. Professional identity theory delves into the internal and social elements that affect career decisions and migration intentions.

Push factors refer to circumstances that drive individuals to depart from their native countries. These may encompass financial difficulties, government unrest, societal challenges, and ecological concerns. Elevated unemployment levels and sluggish economic expansion may compel individuals to pursue improved prospects in various locations (Ochoa-Moreno et al., 2024; Urbański, 2022). Factors such as corruption, inadequate governance, political instability, and restricted freedom of expression play crucial roles in driving these issues (Urbański, 2022; Zaman et al., 2024). Factors such as discrimination and inadequate living conditions can serve as significant motivators for individuals to migrate (Urbański, 2022; Zaman et al., 2024). Natural disasters and unfavorable environmental conditions can compel individuals to relocate (Castelli, 2018). Pull factors refer to conditions that draw individuals to a new location. These may encompass economic opportunities, elevated living standards, educational and career progressions, and social networks. The presence of improved employment opportunities and increased salaries in the host nation is a significant attraction factor (Ochoa-Moreno et al., 2024; Urbański, 2022; Van Der Waal, 2013). Migrants can achieve enhanced quality of life, superior infrastructure, and improved access to services can draw in migrants (Urbański, 2022; Zaman et al., 2024). The potential for advanced education and career development is a major attraction, particularly for students and skilled professionals (Akl et al., 2007; Zaman et al., 2024). Established migrant communities and available social resources in the host country can aid in the migration process (Susilo et al., 2025; Castelli, 2018; Hao, 2012).

Professional identity theory examines how individuals understand and articulate their self-concepts in relation to their professional roles. It encompasses a range of cognitive, motivational, and value-driven traits that shape their approach to the professional landscape and the wider social context (Ivanova, 2008). This theory highlights the complex and ever-changing aspects of professional identity, shaped by various interactions and experiences across different contexts. The concept of professional identity is intricately linked to an individual’s self-concept, encompassing the attributes, beliefs, values, motivations, and experiences that shape their professional persona (Joshy et al., 2025; Slay and Smith, 2011). The process entails the assimilation of professional norms and standards, resulting in individuals adopting the mindset, behaviors, and emotions that are characteristic of their field (Johnson et al., 2023). The development of professional identity is an ongoing journey that starts with career choice and is influenced by experiences throughout training and practice (Brown et al., 2020; Gómez-Gómez et al., 2018). The formation of a professional identity is essential for cultivating a significant understanding of oneself in a professional context, aiding the transition to professional roles (Reissner and Armitage-Chan, 2024). The formation of professional identity is shaped by sociocultural elements and the environment in which individuals engage, encompassing their interactions with colleagues, mentors, and the broader professional community (Best et al., 2022; French and Clarke, 2024).

## 3 Methodology

This study utilized a qualitative case study approach, which involves an in-depth and comprehensive analysis of one or more cases (Creswell, 2009). This method facilitates profound understanding of the processes and outcomes related to a phenomenon (Merriam and Merriam, 2009). This allows for consideration of the context in which the case is situated, aiding in grasping the complexities and nuances of the case (Yin, 2018). In qualitative case studies, data are collected from various sources including interviews, observations, documents, and artifacts. The integration of these diverse data sources significantly enriches this study (Merriam and Merriam, 2009; Yin, 2014).

Purposive sampling was employed to select the participants. Table 2 outlines the demographic profiles of these participants, including administrators, cooperating teachers, faculty members, and pre-service teachers. Purposive sampling was used to identify participants with direct experience with ECE teacher preparation and policy implementation. The intent was to gather rich, contextualized insights from administrators, cooperating teachers, faculty members, and pre-service teachers actively involved in ECE programs. Exclusion criteria included participants who were not affiliated with ECE programs or who had less than 1 year of teaching or administrative experience. Five state-funded Teacher Education Institutions (TEIs) were selected because the Commission officially designates them by the Commission on Higher Education (CHED) as Centers of Excellence in Teacher Education. This ensured that the study focused on institutions that represented the highest quality standards and the effective implementation of ECE programs in the country. The study included 40 key informant interviewees, comprising one administrator, one cooperating teacher, two faculty members, and four pre-service teachers from each university. All the participants were actively involved in pre-service training in early childhood education.

**Table 2:** Demographic profiling of the participants.

University	Position	No. of participants	University academic status of education program	Type of higher education
A	Administrator	1	Center of excellence	State university/government funded
	Cooperating teacher	1		
	Faculty member	2		
	Pre-service teachers	4		
B	Administrator	1	Center of excellence	State university/government funded
	Cooperating teacher	1		
	Faculty member	2		
	Pre-service teachers	4		
C	Administrator	1	Center of excellence	State university/government funded
	Cooperating teacher	1		
	Faculty member	2		
	Pre-service teachers	4		
D	Administrator	1	Center of excellence	State university/government funded
	Cooperating teacher	1		
	Faculty member	2		
	Pre-service teachers	4		
E	Administrator	1	Center of excellence	State university/government funded
	Cooperating teacher	1		
	Faculty member	2		
	Pre-service teachers	4		
	Total	40		

Source: Authors’ tabulated this table.

Key informant interviews (KIIs) entail a systematic approach that encompasses protocols for gathering, presenting, and analyzing data. They highlight the significance of choosing well-informed informants and ensuring adaptability in recruitment approaches (Akhter, 2022). KIIs are qualitative interviews conducted with individuals who have specialized knowledge, experience, or access pertinent to the research topic (Akhter, 2022; Luetke Lanfer et al., 2024). Focus Group Interviews (FGIs) encompass engaging group discussions led by experienced facilitators. The procedure covers the preparation, selection of participants, formulation of interview guidelines, and collection and analysis of data (Chang and Hsu, 2006; Doody et al., 2013). Focus groups typically include 6–12 participants (Smithson, 2015). Each participant was interviewed once and no repeat interviews were conducted. Field notes were taken during and after the sessions to document nonverbal cues and context.

In terms of positionality, the researchers are certified professional teachers, although we do not specialize in early childhood education. Nevertheless, the first author’s doctoral dissertation examines the sustainable migration experiences of Filipino kindergarten teachers in China, while the corresponding author, who acts as an external evaluator, is a researcher who focuses on early childhood education within the Ethiopian context. Before collecting the data, the researchers ensured that they had approval to proceed from the Philippine Normal University Research Ethics Committee (REC) code 2024-236. In addition, this study was part of a larger study. Before beginning data collection, the researchers sought approval from various universities. Focus Group Interviews (FGIs) and Key Informant Interviews (KIIs) were conducted to gather participants’ insights into the preparation, implementation, and assessment of their pre-service Early Childhood Education (ECE) programs. Two FGIs were conducted per university, for a total of ten FGIs across the five participating TEIs. Each comprised six participants—two faculty members and four pre-service teachers—organized homogeneously by role (FGIs 1 for faculty and FGIs 2 for pre-service teachers). All FGIs were conducted onsite within university premises and lasted approximately 2.5 to 3 hr each. KIIs were also held on-site, two per university (one with an administrator and one with a cooperating teacher), totaling ten KIIs, each lasting 1.5 to 2 hr. Each session was facilitated by a two-member research team comprising one facilitator and one documenter, with group size variations determined by the participating universities. A protocol guided the process, outlining the purpose of the study, data privacy, confidentiality, consent procedures, risks and benefits, and data storage. The participants received the interview questions in advance and were encouraged to share and validate their experiences. Each FGI and KII lasted between 2 and 3 hr and took place face-to-face in designated meeting rooms on their respective campuses. Some sessions were held simultaneously, while others were scheduled. The researchers facilitated all the sessions using a prepared interview guide. The interview protocol and questions were displayed on a smart TV or projector during sessions. With the participants’ consent, discussions were audio-recorded, uploaded to a secure online drive, and transcribed for thematic analysis. Potential risks such as discomfort in sharing views and the unintended disclosure of information were mitigated through voluntary participation, informed consent, assurance of confidentiality, and the creation of a respectful discussion environment. None of the participants refused to participate or withdrew from the study.

Data were analyzed thematically following Braun and Clarke's (2006) six-phase approach. Initial codes were generated inductively and then organized deductively around the analytical lenses of the push-pull migration theory (to explain migration intentions) and professional identity theory (to interpret self-perception and role meaning). Cross-case comparisons were conducted across participant groups to strengthen the validity. A key challenge involved distinguishing between overlapping sub-themes (e.g., financial instability as both a push factor and a professional identity issue). This was addressed through peer debriefing among the co-authors and iterative coding verification using ATLAS.ti version 25. In addition, the researcher adhered to the Consolidated Criteria for Reporting Qualitative Research (COREQ), a 32-item checklist designed to ensure transparency and rigor in qualitative studies involving interviews and focus groups. Data collection was continued until thematic saturation was achieved, with no new codes emerging. The participants were invited to review and verify their transcripts for accuracy. Two researchers independently coded the data, and discrepancies were resolved through discussion until a consensus was reached. In addition, an external researcher, the corresponding author, reviewed the themes to ensure constructive alignment with the research questions.

## 4 Findings

Summary of themes are shown in Table 3.

**Table 3:** Summary of themes.

Category	Themes
ECE stakeholders’ perceptions of ECE quality and their professional identity	Perceived quality of ECE programs Career advancement opportunities Financial sustainability of ECE careers Support from educational institutions Societal attitudes toward ECE
Systemic challenges in ECE teacher preparation	Curriculum relevance Resource limitations Inadequate training and support Administrative challenges Perceptions of professional viability
Influencing factors of their migration intentions	Higher compensation Demand from the recruitment agencies Better opportunities abroad & family sacrifices Educational and professional development Decision for knowledge sharing & cultural exchange and learning

### 4.1 What are ECE stakeholders’ perceptions of ECE quality and their professional identity across selected Philippine TEIs

#### 4.1.1 Perceived quality of ECE programs

Stakeholders may perceive the quality of ECE programs differently, which can shape their opinions on the effectiveness of teacher preparation and overall value of ECE as a profession. Concerns about quality can lead to doubts about the viability of a career in ECE. Although there is a framework from the ECCD Council, the challenges lie in its implementation and in fostering synergy among various government agencies, including NGOs, based on the proposed framework. Despite the existence of policies, insufficient emphasis on ECC has led to many daycare centers not being utilized as intended. This stems from the challenges of fostering collaboration among relevant agencies and executing policies aligned with the ECCD framework. According to the statement below, numerous parents, along with communities and barangays, are unaware of ECE’s role of ECE in teacher education and professional practices. Additionally, parents doubt the effectiveness of ECE teachers, as their training is primarily classroom based. Many parents perceive caring for daycare children as akin to babysitting, viewing them as toddlers who are not yet prepared for academic instruction. The confusion surrounding ECE is widespread, and the limited implementation of ECE programs within communities (i.e., barangays) is also lacking.

*"I often hear parents questioning whether our ECE programs truly prepare teachers for the challenges they will face."*

*"There’s a belief that ECE is just babysitting, which undermines the hard work we put into our training."*

#### 4.1.2 Career advancement opportunities

The perception of limited career advancement opportunities within the ECE sector can deter educators from pursuing long-term care. Stakeholders may feel that a lack of clear pathways for growth diminishes the attractiveness of the profession.

*"Many of my colleagues have left for other fields because they see no future in ECE; there are just no promotions available."*

*"It’s disheartening to see talented teachers leave because they feel stuck in their positions."*

#### 4.1.3 Financial sustainability of ECE careers

Concerns about low salaries and financial instability in early childhood education (ECE) can greatly influence stakeholders’ views of the profession’s viability. This often results in educators considering migration to seek better financial opportunities abroad. In day care centers, volunteers or para teachers receive no compensation, meaning that they lack a standard salary or professional fee, which makes the program increasingly unattractive for aspiring ECED professionals. In the Philippine context, the ECCD council and other related agencies pay little attention to day care volunteers and workers, as they perceive early grade teaching as requiring no degree, viewing it as merely play-based and more fun compared to the academic nature of formal education (K-12). However, in many other countries, especially developing countries, governments allocate sufficient budgets for early childhood care, believing that early preparation is crucial for children before they enter formal education. They saw early childhood care as essential for fostering holistic development.

*"I love teaching, but I can’t support my family on this salary. It makes me consider moving to another country."*

*"The pay in ECE is so low that many of us are forced to look for jobs elsewhere, even if we don’t want to leave."*

#### 4.1.4 Support from educational institutions

The extent of support offered by educational institutions, such as mentorship and professional development, can significantly influence perceptions of the quality of ECE. When stakeholders perceive robust institutional support, they may feel assured about the profession.

*"When I started teaching, I had no one to guide me. If there were better support systems, I think more people would stay in ECE."*

*"Institutions need to invest in their teachers; without support, it’s hard to feel competent and valued."*

#### 4.1.5 Societal attitudes toward ECE

Societal perceptions of early childhood education (ECE) as a less prestigious profession can shape stakeholders’ views of their viability. Such negative attitudes may result in a lack of respect for ECE educators, further discouraging them from entering or staying in the field. The implementation design of the Early Childhood Care and Development (ECCD) program clearly indicates that local government units (LGUs), specifically the Department of Social Welfare and Development (DSWD) and barangay local government units (BLGUs), are the primary actors responsible for fulfilling the ECCD mandate at the barangay level. Close monitoring and evaluation of the program are crucial for ensuring the success of the ECCD. However, collaboration among the Department of Education (DepEd), DSWD, and the Department of Health (DOH)—the agencies responsible for properly training and preparing teachers and volunteers to work with children—lacks coordination. Moreover, while DepEd is deeply focused on pedagogy, DOH and DSWD should concentrate on the health and hygiene of children, which is more critical at this stage than academic preparation.

*"People often don’t see ECE as a serious profession, which makes it hard for us to advocate for better conditions."*

*"There’s a stigma attached to being an ECE teacher; many think it’s not a ‘real’ job, which affects how we view our work."*

### 4.2 What are the systemic challenges in ECE teacher preparation among pre-service ECE teachers that shape migration considerations?

#### 4.2.1 Curriculum relevance

The curriculum provided by TEIs may not align with the current needs of the ECE sector, resulting in a disconnection between what is taught and what is required in practice. This misalignment can demotivate educators who are unprepared for real-world challenges. Many day care volunteer teachers or para teachers lack formal teaching preparation or a degree in teaching early grades. They receive special training organized by LGUs through MSWD, and sometimes RHU, on how to handle children. Some first-class municipalities have private day care learning centers, but parents must pay tuition fees for their children. In many cases, these teachers hold degrees or hold a bachelor’s degree. DepEd offers structured training compared with LGU-sponsored training from DSWD and DOH. Many pre-service teachers spend their time within the university and are seldom exposed to local day care centers. Often, they are deployed in city or municipal centers, with very few, if any, visiting remote and far-flung areas. Consequently, even when barangays have day care centers, they are rarely utilized.

*"The courses we take often feel outdated; they don’t reflect the realities of teaching young children today."*

*"I wish we had more practical training instead of just theory. It makes it hard to apply what we learn."*

#### 4.2.2 Resource limitations

Limited resources, including teaching materials, facilities, and technology impede effective teaching and learning. This lack of support can lead to frustration among educators, which affects their motivation and job satisfaction. Although ECED is a play-based approach, it lacks essential materials, such as toys, colorful items, and Lego for children aged 0–4 years. The daycare center’s setup fails to meet both contextualized and minimum international standards for ECED. Teaching and learning resources and materials are not prioritized by the LGU and DSWD.

*"We often have to make do with what we have, which isn’t much. It’s hard to inspire children when you lack basic materials."*

*"The classrooms are overcrowded, and we don’t have enough supplies to engage the kids properly."*

#### 4.2.3 Inadequate training and support

Pre-service and in-service training may not sufficiently equip educators to handle the complexities of early childhood education (ECE). Without continuous professional development, teachers experience feelings of inadequacy and disillusionment. Early childhood is defined as ages 0–8, yet there is unclear curriculum alignment and practice for pre-service teachers, resulting in limited training from universities and day care centers facilitated by DSWD and ECCD representatives. Most training occurs during the practicum, which is confined to DepEd classrooms from kindergarten to Grade 3. There is a lack of training from daycare centers for children aged 0–4, organized by the DSWD and ECCD council representatives. Instead of conducting practicums or teaching at daycare centers based on the ECED curriculum, these activities often become community extensions, a separate function of the university not included in the ECED curriculum, but required for accreditation purposes.

*"After graduation, I felt lost. There was no mentorship or guidance to help me transition into teaching."*

*"We need more workshops and training sessions to keep up with best practices in early childhood education."*

#### 4.2.4 Administrative challenges

Encountering bureaucratic obstacles and lack of administrative support can make the work environment challenging. Educators may feel undervalued and demotivated when their concerns remain unaddressed. The absence of a university-LGU-BLGU Memorandum of Understanding (MOU) is notable. Additionally, compliance with the CHED’s requirement for pre-service teachers’ community immersion necessitates obtaining permission from the students’ affairs office. Proper coordination between ECCD representatives and DSWD (BNS and BHW) is essential.

*"It feels like the administration doesn’t listen to us. We have ideas for improvement, but they go unheard."*

*"There’s so much red tape that it’s hard to implement changes that could benefit our students."*

#### 4.2.5 Perceptions of professional viability

The perception that ECE is not a viable career option discourages educators from fully committing to their roles. Concerns about job security and career advancement can lead to a lack of motivation and increased migration intention.

*"Many of my peers are considering leaving the profession because they don’t see a future in it."*

*"I love teaching, but I worry about my financial stability. It’s hard to stay motivated when the pay is so low."*

### 4.3 Based on these systemic challenges and perceptions of ECE quality, what factors contributed to their migration intentions to leave the Philippines?

#### 4.3.1 Higher compensation

ECCD teachers are attracted to countries that offer significantly higher salaries and benefits than those available in the Philippines. One of the teachers said,

In addition, for example, the college of education produces trained early childhood educators, but what draws them away are opportunities in other countries for higher pay because they will go abroad. So none are left. Skilled pre-service teachers or teachers who could ideally impart their knowledge to the betterment of the curriculum or education system. Hopefully, more opportunities will be given to early childhood educators so that leaving the country no longer seems to be the only or best option for them. Because the compensation [offered abroad] is really generous with so many benefits, and considering such things, hopefully the government… hopefully there are no corrupt politicians anymore now.

#### 4.3.2 Demand from the recruitment agencies

Many teachers shared that many of their students were recruited directly from agencies. The agencies look for possible employment abroad and persuade them to have a better overall quality of life, including access to healthcare, education, and social services, which are often more robust in other countries. One of the faculty members shared:

*Mostly, they are contacted by agencies, because last time, I remember, the agencies really came here. And then they are contacted, saying that when you graduate and pass the licensure examination, you will be directly hired. So, it's a sad reality that we have free education here in West Visayas. We were trained and equipped with knowledge, but then, on the other hand, we are imparting our knowledge there [abroad] because we are practicing it in other countries. But sometimes there are others also who learned the systems there, and they, as well, helped the university or the college share that knowledge. Because there are also seniors who share their curriculum. It shouldn't be allowed, but it seems to be also shared. Such as strategies that can better improve our education system, most especially in education.*

#### 4.3.3 Better opportunities abroad

Many individuals perceive migration as a pathway for better opportunities, including higher salaries and improved living conditions. The allure of foreign employment often outweighs the challenge of leaving home.

*Interviewee*

*It's difficult to choose the Philippines.*

*Interviewer*

*In one year, they already bought a car. The next year, a house, the a farmland.*

*Interviewee*

My aunt, ma'am, was already a master teacher in high school in Pangasinan in Dagupan. So, she saw the [opportunities/prospects] for ECE in Canada. She started as a cleaner in Canada while she was studying for ECE, ma'am, because she was a high school science major here, so she also needed to take ECE. Oh now, her whole family is there already. She traded what she had here. She was an ECE teacher here. It's true, ma'am. What can we do against money?

##### 4.3.3.1 Family sacrifices

The decision to migrate often involves significant family sacrifice. Individuals express a desire to support their families financially, even if it means leaving loved ones behind.

*It's more of a decision. I am the one who will leave for the family. But I don't want to leave my parents.*

#### 4.3.4 Educational and professional development

Migration is often linked to the pursuit of education and professional development. Graduates seek opportunities abroad to enhance their skills and gain international experience, which they believe will benefit their career.

*Because they want to get into this program. Of course, ma'am, we are government-funded, tuition-free, which is a big deal for them*

#### 4.3.5 Decision for knowledge sharing

There is concern about brain drain, where skilled professionals leave the country for better opportunities abroad, resulting in a shortage of qualified individuals in the local education system.

*"Also, for example, the College of Education is producing trained early childhood educators, but the ones who are getting them are those from other countries because they will go abroad. So, no one is left. The skilled pre-service or teachers who could have imparted their knowledge for the betterment of the curriculum or the education system. So, I hope the early childhood educators are given more opportunities so that it won't be an option for them to go abroad anymore. Because the compensation being given, there are really so many benefits in the Philippines. And considering those things, I hope the government… I hope there are no more corrupt politicians now."*

##### 4.3.5.1 Cultural exchange and learning

Migration also facilitates cultural exchanges and learning. Individuals who migrate often bring back knowledge and experience to enhance their local educational practices. The multifaceted nature of migration encompasses aspirations for better opportunities, familial responsibilities, professional growth, and implications for local educational systems.

*"But sometimes there are also others who learned the systems there, and they also helped the university or the college to share. Because there are also seniors who share their curriculum. It shouldn't be allowed, but it seems they do share it. Like strategies to better improve our education system, especially in education."*

## 5 Discussion

Table 4 illustrates that the migration of Filipino ECE teachers is not solely a response to economic or structural factors, but is also deeply rooted in their sense of identity. The push-pull migration theory sheds light on the reasons teachers leave, focusing on external factors such as pay, recognition, and opportunities. Professional Identity Theory examines how teachers perceive and internalize these factors, thereby influencing their professional self-image. By combining these theories, we gain a comprehensive perspective that migration is both a response to systemic inequalities and an expression of professional autonomy. These findings underscore the need for ECCD reforms that bolster teacher identity through coherent policies, professional recognition, and fair working conditions, along with current policy analyses that emphasize the importance of teacher quality and retention in the Philippine ECCD system (Ulep et al., 2023, 2024).

**Table 4:** Mapping of study findings in relation to push–pull migration theory and professional identity theory.

Major findings / themes	Explanation from findings	Push–pull migration theory (Lee, 1966)	Professional identity theory (Beauchamp and Thomas, 2009)
1. Systemic challenges in ECE preparation	Teachers described underfunded ECE programs, low salaries, lack of promotion, and inadequate policy implementation in the Philippines.	Push factors: Structural inequities (low pay, poor support, limited growth) “push” ECE teachers to seek better working conditions abroad.	These challenges erode professional identity, diminishing teachers’ self-efficacy and sense of professional worth.
2. Attraction to overseas opportunities	Respondents perceived migration as a pathway for stability, higher income, and international professional recognition.	Pull factors: Overseas employment provides financial rewards, professional autonomy, and career development—serving as strong “pull” forces.	Migration represents a means of reconstructing identity, restoring dignity and recognition that were absent locally.
3. Social recognition and professional respect	Participants expressed that ECE teachers are undervalued in the Philippines compared to abroad.	Limited recognition acts as a push factor intensifying dissatisfaction with local conditions.	Recognition abroad validates teachers’ expertise, thus strengthening professional identity and self-esteem.
4. Institutional preparation and mismatch	The study found a gap between teacher preparation and labor demands, leading to professional frustration and misalignment with ECCD expectations.	The lack of systemic support increases migration intention, as teachers perceive limited opportunities for growth.	Misalignment between training and practice weakens the formation of stable professional identity during pre-service years.
5. Migration as a form of agency and professional renewal	Teachers interpret migration as a way to reclaim agency and pursue self-development.	Migration becomes both a response to push–pull dynamics and an active decision for self-fulfillment.	Migration is viewed as a process of identity reconstruction, where teachers negotiate belonging and meaning in new educational contexts.
6. Policy gaps and teacher retention	Findings highlight weak coordination between CHED, DepEd, and ECCD Council in supporting ECE professionals.	Structural limitations in domestic policy represent contextual push factors driving mobility.	Policy neglect undermines teachers’ collective professional identity, reducing commitment to the local system.

The authors tabulated this table.

Based on the findings, Early Childhood Education stakeholders in selected Philippine Teacher Education Institutions perceive ECE quality as hampered by implementation gaps and resource limitations, and their professional identity is undermined by limited career advancement, financial instability, inadequate institutional support, and societal attitudes viewing ECE as low-status “babysitting.” While pre-service ECE training is acknowledged to effectively preparing future educators, boost their confidence in the field, and equip them with practical skills for real-world applications, as evidenced by the migration of ECE graduates to foreign jobs, there is a distinct need for more proactive and hands-on training, including immersion in the actual environment of young children (Ancho et al., 2023). The gap in providing experiential learning for pre-service ECE teachers, particularly infants and toddlers, largely depends on the availability of Barangay Child Development Centers (CDCs) to accept apprentices. Generally, Barangay CDCs in the Philippines suffer from inadequate facilities and infrastructure, with some even operating outside of common barangay hall premises. Only a few Barangay CDCs have dedicated canteens, libraries, multipurpose halls, kitchens, and clinics (Abulon, 2010). Furthermore, effective apprenticeship in ECE requires a competent mentor such as a Child Development Teacher (CDT) or Child Development Worker (CDW). However, local government units (LGUs) face financial constraints in hiring qualified CDTs or CDWs, resulting in most workers being deployed in Barangay CDCs as volunteers who are not necessarily trained in early childhood education or do not hold a degree in ECE (Biana et al., 2021).

Regarding the second research question, the systemic challenges shaping migration considerations include curriculum misalignment with practical needs, severe resource limitations, inadequate training (especially for ages 0–4), bureaucratic administrative challenges, and the perception that ECE is not a valued profession. Systemic challenges persist, prompting pre-service ECE teachers to consider migration. Despite the policy standards and guidelines established by the Philippines’ Commission on Higher Education (CHED) for implementing a bachelor’s degree in ECE, there remains a lack of effective supervision and coordination in strengthening the nation’s teacher education system. However, high-quality institutions that offer ECE programs are limited. Only a small fraction of teacher education programs (36 of over a thousand) have achieved COE status, and only 38 have been designated as Centers of Development (COD), highlighting the need for more proactive measures to incentivize quality (Generelao et al., 2022). Moreover, ECE teachers encounter significant challenges such as a lack of necessary resources, underscoring the need for improved training and support (Kim et al., 2024). The education system also faces challenges, such as overcrowded classrooms, insufficient teaching staff, limited resources, inadequate salaries, and corruption. These factors hinder effective teacher training (Chua, 2019). Notably, in the Philippines, no Teacher Education Institution (TEI) in Mindanao, particularly in BARMM and Region 12, has met the CHED requirements to become a COE in Teacher Education (CHED, 2018). This situation underscores the quality of pre-service ECE programs offered in the Philippines, reflecting regulatory bodies’ lack of oversight. Limited funding for early childhood care and development leads to insufficient program resources, hindering Teacher Education Institutions (TEIs) from deploying pre-service ECE teachers. Similarly, in Ethiopia, ECE teachers face the challenges of limited monitoring, insufficient parent involvement, ineffective school leadership, poor staff-to-child ratios, and inadequate healthcare standards (Taddese et al., 2025).

Early childhood education (ECE) remains a low-status profession both globally and in the Philippines, with significant implications for teachers’ professional identity. ECE teachers perceive their profession as having a lower status than other occupations, and in earlier decades (Aydin et al., 2015), often due to low salaries, poor working conditions, and a lack of public recognition (Abulon, 2010; Moloney, 2010; Taddese et al., 2025). These conditions contribute to a fragile sense of professional worth, pushing ECE graduates to pursue jobs abroad or change their careers once they leave the country. Efforts to uplift the profession through improved pre-service education, ongoing professional development, and support for educators working abroad have shown promise (Ancho et al., 2023). However, inconsistencies persist between the critical social role of ECE and its limited recognition it receives (Hargreaves and Hopper, 2006; Vujičić et al., 2014). Without addressing these systemic challenges, the professional identity of ECE teachers will remain undermined, affecting not only workforce sustainability but also the quality of early childhood education.

Lastly, the third research question, ECE stakeholders’ migration intentions are primarily driven by the pursuit of significantly higher compensation, active recruitment by foreign agencies, better professional opportunities and living standards abroad, the need to support families financially despite personal sacrifice, and brain circulation. Despite the vital role of ECE teachers in national development, many experience economic dissatisfaction due to low salaries, poor working conditions, and limited career advancement opportunities. Compared to neighboring countries, the Philippines offers some of the lowest wage rates for educators, making overseas employment a compelling alternative (Mendoza et al., 2013). Particularly for teachers in the Barangay CDC, a mere Php5,000.00 honorarium is given per month (approx. $90) (Abulon, 2010), which clearly does not qualify as decent wage. The low wages and poor working conditions of ECE teachers and graduates have become an opportunity for recruitment to work abroad. Foreign agencies actively recruit Filipino teachers, offering higher salaries, better living standards, and access to advanced educational environments. Countries such as the United States, the United Kingdom, and Japan attract teachers through streamlined recruitment processes, stable institutions, and opportunities for professional growth. These “pull” factors are especially attractive given the “push” of domestic challenges, including job skills mismatches, bureaucratic inefficiencies, and diminishing professional autonomy (Tabuga, 2018). The economic need to support families is at the core of such decisions. Many teachers view migration as a strategic move to improve their families’ financial well-being, often sending remittances that support education, healthcare, and daily living expenses (Castro-Palaganas et al., 2017). While personal and social sacrifices such as separation from loved ones are acknowledged, the promise of greater financial stability and professional recognition continues to motivate educators to seek employment abroad (De Jong and Fawcett, 1981; Tsang and Lowe, 2018).

## 6 Implications for the theory, policy, and practice

This study demonstrates that push factors (systemic deficiencies in ECE preparation, low pay, societal stigma) and pull factors (higher compensation, better opportunities abroad) are deeply intertwined with professional identity formation, moving beyond purely economic motivations to include professional dignity, efficacy, and societal recognition as critical drivers of teacher migration. The study recommends that pre-service teachers have an additional diploma and certification in Early Childhood Care and Development from TESDA. For example, understanding personal hygiene, nutrition, safety, and well-being toward holistic child development. In addition, educate the community to change their mindset about the role of pre-service, in-service, and volunteer teachers/para-teachers in daycare centers. Teachers are regarded as the cornerstone of lifelong learning and the development of the future workforce rather than merely being seen as babysitters, thereby upholding the dignity of their professional identity.

Furthermore, it highlights that, in the Philippines, systemic challenges and low societal valuation significantly undermine professional identity in ECE. The perceived inability to establish a viable and respected professional identity locally serves as a strong push factor, whereas the prospect of enhanced professional status and development abroad acts as a pull factor. The study recommends institutionalizing the ECE program and securing government funding while seeking support from public-private partnerships (PPP). To maintain curriculum relevance, address resource limitations, provide adequate training and support, and overcome administrative challenges. These efforts could help to retain ECE teachers and prevent their migration.

For the Philippine government, particularly agencies such as the Department of Interior and Local Government (DILG), Department of Health (DOH), Department of Education (DEPED), Department of Social Welfare and Development (DSWD), Commission of Higher Education (CHED), National Nutrition Council (NNC), and Technical Education and Skills Development Authority (TESDA), it is recommended to offer fair compensation to teachers handling Early Childhood Education. Strong support systems should be established for training skills and professional development. Additionally, knowledge sharing and cultural exchange opportunities should be provided for ECE pre-service teachers to intern abroad, thereby strengthening school-community-industry-university partnerships locally and abroad, and meeting global standards. To contextualize the newly established law in Early Childhood, it is advisable to implement a well-defined and adequately funded career progression ladder within the ECE sector. Additionally, school personnel should be provided to assist teachers with auxiliary tasks. Infrastructure and facilities should be contextualized and appropriate. Furthermore, ECE teachers should undergo extensive immersion or internships to fully comprehend the ECE teaching context.

## 7 Conclusion

This study aimed to examine the systemic challenges in early childhood education (ECE) teacher preparation and how these shape the migration intentions of ECE stakeholders, including administrators, faculty, and pre-service teachers, across selected teacher education institutions. The findings reveal that ECE programs are perceived to suffer from low quality, limited career advancement opportunities, inadequate institutional support, and societal undervaluation. These challenges manifest as push factors that drive educators to seek professional opportunities abroad, while pull factors, such as higher compensation, structured career progression, and greater professional recognition, encourage outward mobility. In terms of policy relevance, the findings underscore the need for the Commission on Higher Education (CHED) to review and revise ECE curricula to better align teacher preparation with workforce realities, emphasizing competencies in early childhood care and development and inclusive pedagogy. Similarly, agencies such as the Department of Labor and Employment (DOLE) and the Department of Education (DepEd) should implement targeted teacher retention programs, ensuring equitable compensation, effective workload management, and structured professional development to mitigate migration pressure. Collaboration with the Department of Social Welfare and Development (DSWD) and the Technical Education and Skills Development Authority (TESDA) is also vital for strengthening early childhood teacher certification and career pathways, in line with the Early Years Act of 2013 and the ECCD Council policy reforms. Theoretically, this study advances scholarship by integrating push-pull migration theory and professional identity theory, offering a dual-lens perspective of teacher migration as both a structural and an identity-driven process. Migration decisions among ECE teachers are not merely economic responses to unequal labor markets, but also expressions of professional identity reconstruction, in which educators seek dignity, recognition, and a renewed sense of purpose in their profession. However, the study is limited by its focus on selected institutions and qualitative scope, which may constrain its generalizability to all ECE contexts in the Philippines. Future research should adopt mixed-method or longitudinal designs to trace the career trajectories of ECE graduates, document actual migration outcomes, and compare teacher retention strategies across the Global South. Sustainable teacher mobility requires building a resilient ECE ecosystem that values teachers as central to early learning and national development, while strengthening inter-agency collaboration to ensure that teacher migration becomes an empowered choice rather than a forced necessity.

## Data Availability

The raw data supporting the conclusions of this article will be made available by the authors, without undue reservation.
